# Supplementary material for: Changes of Target Essential Trace Elements in Multiple Sclerosis: A Systematic Review and Meta-Analysis
Source: Biomedicines. 2024 Jul 17;12(7):1589. doi: 10.3390/biomedicines12071589 (PMC11274787; doi:10.3390/biomedicines12071589)
Supplement: Supplementary file 1 [file biomedicines-12-01589-s001.zip › biomedicines-3055421-supplementary.pdf]

PRISMA 2020 for Abstracts Checklist

Section and Topic	Item #	Checklist item	Reported (Yes/No)
**TITLE**			
Title	1	Changes of target essential trace elements in multiple sclerosis: a systematic review and meta-analysis	No
**BACKGROUND**			
Objectives	2	Although the first papers on the potential role of trace elements in MS were done in the 70s, for five decades it remains unknown whether trace elements can be part of this heterogeneous neurological disease. To arrive at a potential solution, we conducted a systematic review and meta-analysis to clarify if most of the studied essential trace elements (Fe, Co, Mn, Zn, Cu, and Se) have the potential to be associated with MS.	No
**METHODS**			
Eligibility criteria	3	Our inclusion criteria included original case-control, cohort and cross-sectional studies studies in which the levels of the elements of interest (Zn, Fe, Co, Cu, Mn, and Se) were reported in the specified clinical matrices by both MS and control subjects.<br>Exclusion criteria were studies in which the diagnosis of MS was not confirmed, studies with cases and controls not from the same residence, studies with age- and sex-atypical cases and controls, studies that reported additional pathologies besides MS, non-English language studies, studies with insufficient numerical data, and studies with extremely abnormal values of the analyzed elements. We only included studies that contained results where the mean ± standard deviation (SD) or standard error (SE) was reported. We also used data with a different numerical value from which the SD can be calculated. We excluded studies that reported only the mean without SD or SE, or studies in which only graphs without numerical values were shown.	No
Information sources	4	SCOPUS, Science Direct, PubMed, Google Schoolar	No
Risk of bias	5	Egger’s regression test and Begg and Mazumdar’s rank correlation test.	No
Synthesis of results	6	Pooling of the data using the random effects model, Hedges's g, Individual study effect sizes, Relative weights and standard residuals for each study, heterogeneity.	
**RESULTS**			
Included studies	7	84 studies, 4461 cases / 3888 controls, totally 8349 participants	No
Synthesis of results	8	Zn: 1308 cases/1096 controls, pooled size effects p= 0.012, significantly decreased in cases<br>Fe: 1096 / 595, pooled size effect p=0.007, significantly decreased in cases<br>Co: 405 / 358, pooled size effect, p=0.326, N.S.<br>Cu: 1143 / 1031, ooled size effect, p=0.002, significantly increaesd in cases<br>Mn: 460 / 453, pooled size effect, p=0.590, N.S.<br>Se: 536 / 355, pooled sze effect, p=0.351, N.S.	No
**DISCUSSION**			
Limitations of evidence	9	Although strict criteria were used to appropriately include individuals and exclude papers with very high levels of investigated trace elements in clinical matrices, we were unable to separate all participants by gender, age, or residence, as the dimensionality required for reliable meta-analysis was lost. One of the primary challenges in elucidating the etiological role of trace elements in MS is the lack of access to the most authoritative clinical matrices to either confirm or refute a causal relationship. This primarily pertains to the inability to collect brain tissue from patients with MS due to the impracticality of surgical or biopsy procedures. The situation is further complicated by the fact that the exact timing of MS onset remains unknown; symptoms emerge at varying ages.	No
Interpretation	10	In summary, the results indicate that these trace elements could be either a risk factor or a consequence of Fe and Zn loss as a result of disease, indicating that targeted supplementation should be considered in patients with MS. On the other hand, a high Cu level indicates a burden on the bloodstream of MS patients and could be considered from an etiological point of view	No

PRISMA 2020 for Abstracts Checklist

Section and Topic	Item #	Checklist item	Reported (Yes/No)
**OTHER**			
Funding	11	Ministry of Science, Technological Development, and Innovation of the Republic of Serbia, Contract Numbers 451-03-66/2024-03/200288 and 451-03-66/2024-03/200007.	No
Registration	12	PROSPERO ID: CRD42024524428	No

From: Page MJ, McKenzie JE, Bossuyt PM, Boutron I, Hoffmann TC, Mulrow CD, et al. The PRISMA 2020 statement: an updated guideline for reporting systematic reviews. BMJ 2021;372:n71. doi: 10.1136/bmj.n71

For more information, visit: http://www.prisma-statement.org/
